# openBEB: open biological experiment browser for correlative measurements

**DOI:** 10.1186/1471-2105-15-84

**Published:** 2014-03-26

**Authors:** Chandrasekhar Ramakrishnan, Andrej Bieri, Nora Sauter, Sophie Roizard, Philippe Ringler, Shirley A Müller, Kenneth N Goldie, Kaloyan Enimanev, Henning Stahlberg, Bernd Rinn, Thomas Braun

**Affiliations:** 1 Center for Cellular Imaging and NanoAnalytics (C-CINA), Biozentrum, Universität Basel, Basel, Switzerland; 2 Center for Information Sciences and Databases (C-ISD), Department of Biosystems Science and Engineering, Swiss Federal Institute of Technology Zurich, Basel, Switzerland; 3 Swiss Institute of Bioinformatics, Biozentrum, Universität Basel, Basel, Switzerland; 4 Laboratory of Physical Chemistry of Polymers and Membranes (LCPPM), École Polytechnique Federale de Lausanne, Lausanne, Switzerland

## Abstract

**Background:**

New experimental methods must be developed to study interaction networks in systems biology. To reduce biological noise, individual subjects, such as single cells, should be analyzed using high throughput approaches. The measurement of several correlative physical properties would further improve data consistency. Accordingly, a considerable quantity of data must be acquired, correlated, catalogued and stored in a database for subsequent analysis.

**Results:**

We have developed openBEB (open Biological Experiment Browser), a software framework for data acquisition, coordination, annotation and synchronization with database solutions such as openBIS. OpenBEB consists of two main parts: A core program and a plug-in manager. Whereas the data-type independent core of openBEB maintains a local container of raw-data and metadata and provides annotation and data management tools, all data-specific tasks are performed by plug-ins. The open architecture of openBEB enables the fast integration of plug-ins, e.g., for data acquisition or visualization. A macro-interpreter allows the automation and coordination of the different modules. An update and deployment mechanism keeps the core program, the plug-ins and the metadata definition files in sync with a central repository.

**Conclusions:**

The versatility, the simple deployment and update mechanism, and the scalability in terms of module integration offered by openBEB make this software interesting for a large scientific community. OpenBEB targets three types of researcher, ideally working closely together: (i) Engineers and scientists developing new methods and instruments, e.g., for systems-biology, (ii) scientists performing biological experiments, (iii) theoreticians and mathematicians analyzing data. The design of openBEB enables the rapid development of plug-ins, which will inherently benefit from the “house keeping” abilities of the core program. We report the use of openBEB to combine live cell microscopy, microfluidic control and visual proteomics. In this example, measurements from diverse complementary techniques are combined and correlated.

## Background

Systems biology aims to identify and quantify the molecular components of dynamic biological networks, determining interactions between the various players and integrating the resulting information into system models [1]. This research necessitates the use of an ensemble of correlative measurement technologies. Ideally, data should be acquired from groups of elementary samples, such as single cells, using high throughput technologies, in order to disentangle *biological noise* due to the stochastic nature of interaction networks [2]. An experimental environment of this type will generate large quantities of heterogeneous but related data. This presents many challenges, including the key problem of tracking and integrating measurements made on a series of related samples across diverse technological platforms.

A number of software tools are available to handle data originating from high throughput experimental set-ups. These are technique specific. Examples are, *Omero* for light microscopy [3], *Leginon* for electron microscopy (EM) [4] and *PRISM* for high-throughput proteomics [5]. Difficulties arise when several instruments and/or complex (automated) preparation steps are required for the research, as is often the case in a micro-fluidic pipeline. One way to create a multi-instrument solution would be to amalgamate the domain-specific software systems. The disadvantage is that combinatorial problems caused by required interaction between and coordination of the individual software packages, will increase rapidly with the number and complexity of the technologies involved. Furthermore, the correlation of individual datasets in relation to space and time will become progressively more difficult.

Flexible data management systems such as *openBIS* (open Biological Information System) [6] offer a partial solution, providing scalable data storage and retrieval, metadata integration and searching, and data source tracking. Although the platform-independent, web-based graphical user interface (GUI) of openBIS allows user management, authorization and configurable database browsing, it does not allow in depth data handling and does not support direct instrument control. These shortfalls are overcome by the new software presented in this paper, openBEB (open Biological Experiment Browser).

The requirements to be met become apparent when the following typical example is considered (see also results and discussion): Eukaryotic cells growing in miniaturized Petri dishes and are subjected to pulse chase experiments. During the experiment, the cells are observed by time-lapse light microscopy (LM). At specific time points, individual cells are lysed and prepared for further analysis by EM. Subsequently, specific features of the images, e.g., fluorescence signals detected by LM, are tracked over time. This scenario has three requirements: (i) Data acquisition and instrument control must be tightly integrated. (ii) Various data types must be collected and handled, e.g., image data and time-resolved “wave” data. (iii) The individual steps of the experiment must be correlated in space and time, e.g., EM data of an individual cell must be assigned and correlated to series of time-lapse LM images. OpenBEB provides a flexible, data-type agnostic core framework that performs the tedious “house keeping” tasks demanded, such as data management and the creation and maintenance of a unified hierarchical coordinate (HC) system. The latter establishes the relationships between experimental results that have to be retained in multi-scale space and time, a fundamental requirement for any correlative measurement. Furthermore, openBEB provides a plug-in manager that supports plug-ins for data-type specific tasks and instrument control. An internal macro system allows the control and coordination of these individual technology-specific modules. Furthermore, plug-ins can be used to connect openBEB to databases such as openBIS, facilitating data storage and synchronization.

OpenBEB furnishes the *end-user* with an environment for instrument control, data acquisition, visual inspection, advanced visualization, annotation, information correlation and metadata management. Of advantage for the *developer*, the software architecture allows the rapid integration of new instrument-specific modules, facilitating the use of correlative methods for systems biology, e.g., complex micro-fluidic set-ups.

## Implementation

Central goals of openBEB are to provide both an environment allowing the fast integration of correlative measurements and a platform allowing the rapid development of control-software. The extensible openBEB framework is implemented in LabView using object-oriented “loose coupling” principles. The latter is achieved via a plug-in structure. The programming language, G, makes it possible for researchers with little or no programming experience to develop their own extensions with minimal effort. The extensive libraries of the LabView environment for instrument control, data acquisition and signal processing are optimal for the implementation of the automatic data acquisition essential to realize high throughput.

**Figure 1:**
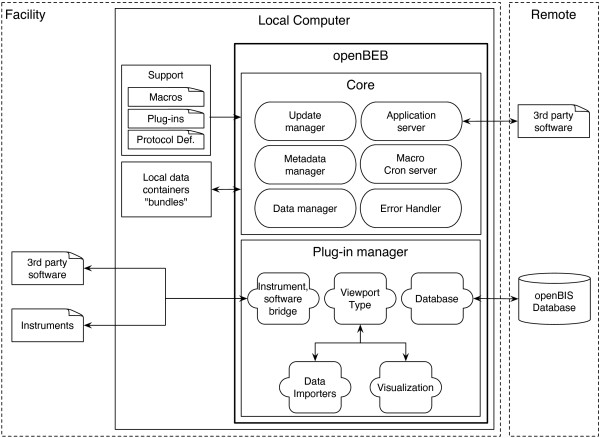
**Overview of the openBEB software.** For *end-users*, openBEB acts as a link between experimental biology and database systems. It is designed to facilitate data acquisition, browsing, annotation and synchronization with databases. For *developers*, it provides an environment for the fast development of data-acquisition and instrument control plug-ins, providing a core framework for data-management and coordination of different modules by a unified macro language. OpenBEB runs locally and consists of a core program and a plug-in manager. Plug-ins are dynamically loaded during start-up. The core program maintains a local bundle, i.e., a container structure in the file system for raw-data, metadata and cache files. All files (except the cache files) are readable by standard programs, can be browsed in the file system, and can be directly accessed by other local programs. The core program can be controlled by a graphical user interface (GUI) and via macros and a TCP-based protocol. The update manager of openBEB automatically updates an application support folder containing metadata definition files and plug-ins. Plug-ins are loaded and coordinated by the plug-in manager. The open architecture of openBEB allows plug-ins to be written to handle different types of data (data-type plug-ins, e.g., for images or simple xy-scatter data). Data-importers or plug-ins for data visualization (called viewports) can be loaded. The plug-in manager also provides a unified interface for instrument control plug-ins facilitating the rapid development of instrument control software. Database synchronization is also achieved using plug-ins; the standard openBEB installation is designed to communicate with the openBIS system [6]. The error handler provides a logging and error-processing tool for the core program as well as for the plug-ins.

OpenBEB consists of two parts (Figure 1): (i) A static core program, responsible for data and metadata handling and the coordination of different modules. (ii) A dynamic plug-in architecture. Plug-ins supply the case-specific functionality of openBEB, and are dynamically loaded by the plug-in manager during program start-up. They provide the tools of the browser, such as data-type specific libraries, importers, data visualization modules (called viewports), instrument controllers as well as database bridges, e.g., for openBIS. This modular architecture allows maximum flexibility and scalability.

### Core program

A queued message state machine drives the core program (Figure 1). The central message queues can receive commands from several sources, such as the GUI, the *macro-parser* or the *application server* via a TCP based protocol. The latter allows platform-independent communication with other software packages or remote instrument controllers and computers (details are provided on the application home-page). Complex operations can be controlled by text-based macros that are located in the *application support* folder and can be adjusted by users if needed (*Macro and Cron server*). The *data manager* organizes the import and management of data in a data-agnostic manner and can handle arbitrary types of data. The data-types themselves are defined in plug-ins as described below. Furthermore, the *data manager* of the core program maintains one or more local containers for data and metadata called “bundles”. Bundles rely on a hierarchical file structure and can be directly accessed by any program without the need of the openBEB software. To avoid conflicts with automated processes, access to bundles or data collections they contain via the GUI can be blocked. Data-management tasks are performed centrally by the *data-manager*, which can be reserved by a process, e.g., a macro (see below). The data manager calculates MD5 checksums during data import as a control measure to check the integrity of the raw data. OpenBEB never changes the raw data. Furthermore, the data manager allows transparent, lossless data compression by the ZIP algorithm [7]. The *metadata manager* organizes the metadata, which are stored in an xml format. The metadata includes the embedded metadata of the raw data, which can be quite extensive for some data types. In addition, the *metadata manager* supports pre-defined metadata annotations called “protocols”, based on a controlled vocabulary. These predefined protocols are located in the *application support* folder. This support folder is automatically updated by the *update manager* during openBEB start-up, keeping all components, e.g., plug-ins and protocol definition files, up-to-date with a server-side repository. Last but not least, a central *error handler* and logging system monitors all activities of both the openBEB core program and the plug-ins.

#### Macro control and modules

OpenBEB includes a simple macro interpreter, which also allows unified control of the modules provided by plug-ins. The macro language is primarily an inter-module processing and coordination system. It supports both the use of variables and branching and looping, provides primitives for graphical user interaction (dialogs and user input), and supports communication between modules. A TCP based application server is integrated to handle applications of greater complexity or to provide an interface to existing software.

Importantly, the macro interpreter has an open modular architecture. Every plug-in can provide one or several modules that can be addressed by the macro interpreter. Every module implicitly consists of a queued state-machine architecture and runs independently; this is particularly important for instrument control. A module is associated with a name and must follow specific rules for command and error handling. Furthermore, a minimum set of commands, e.g., for process coordination, must be supported. The coordination of different modules is achieved using synchronization primitives that are available in the macro language.

**Figure 2:**
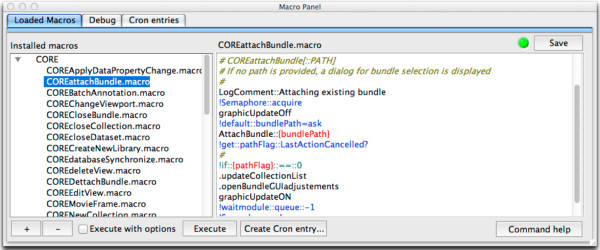
**OpenBEB macro panel.** The macro panel allows the creation and editing of macros controlling the core program and the plug-in modules. Furthermore, it provides a GUI to control the timed execution of tasks by a cron-like server.

A macro panel (Figure 2) simplifies the creation and editing of macros as well as task handling; start, execution and scheduling (“cron”). The debug menu provides information about the last run of the selected macro, such as the variables used. The “cron “ tab entry configures a cron-like server (part of the core program) for the time specific execution of macros. This is useful to trigger macros to prepare instruments ahead of usual working times, e.g., to carry out routine cleaning procedures or to activate temperature controls.

#### Local repository

OpenBEB maintains a local repository for raw-data, metadata and cache files. This is accessible by standard tools of the operating system. Standard formats are used, except for the instrument dependent raw-data and the cache files. The local repository can be browsed by a file-browser, such as the Macintosh Finder or Windows Explorer, therefore, the entire contents are accessible to 3^rd^ party software.

**Figure 3:**
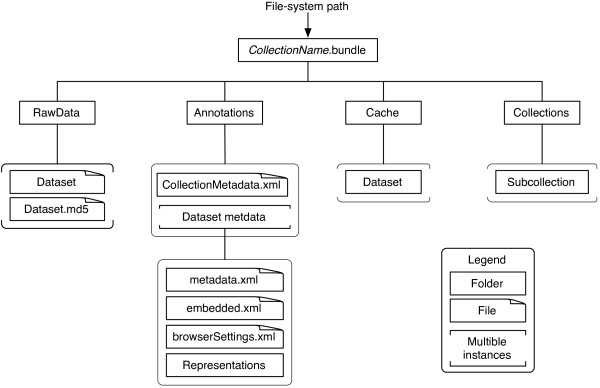
**Data organization of the local repository maintained by openBEB.** Data are organized hierarchically in collections. The uppermost level (root collection) is called a bundle. Bundles contain collections, and collections can contain other collections: The collection root is denoted *collectionName*.bundle, where *collectionName* is the bundle name. A bundle defines the file system location (*file-system path*) where the collection hierarchy is stored. A collection contains four folders: Folder one, *RawData*, contains the raw-data. The raw data is never changed by openBEB, however, openBEB calculates an MD5 check sum to monitor the data integrity and allows data compression by a lossless algorithm. Folder two, *Annotations*, contains metadata information, preview and graphically annotated representations of the data. It also contains the embedded metadata of the raw files in a standardized xml format. Folder three, *Cache*, contains cache information. This information stores cache files for faster browsing or stores processing results. All of the cache information is redundant and can be recalculated at any time from the information contained in folders one and two. Consequently, the information in the cache folder is not synchronized with the database. Folder four, *Collections*, is a container hosting sub-collections, allowing an arbitrary hierarchical, tree-like structure of collections to be built.

OpenBEB organizes data in “collections”, i.e., containers that can but do not have to host a series of raw datasets and associated metadata files. In the simplest case, a collection only consists of a metadata file. Collections can contain other collections, and can thus be organized in a hierarchical structure. The upmost collection, the root of the collection hierarchy, is called a “bundle”. A bundle is a collection associated with a path in a file-system. The structure of the openBEB bundle is outlined in Figure 3. Several bundles can be attached to and maintained by an openBEB instance.

Note, that the collection hierarchy of a bundle does not necessarily reflect the workflow of experiments. However, the workflow is preserved in the database system, e.g., openBIS [6]. A bundle is a work-snapshot and can combine collections from different spots in the workflow, e.g., a bundle can contain collections of original data obtained by different techniques and collections that amalgamate the results.

#### Annotating metadata, hierarchical coordinate system and graphical annotations

**Figure 4:**
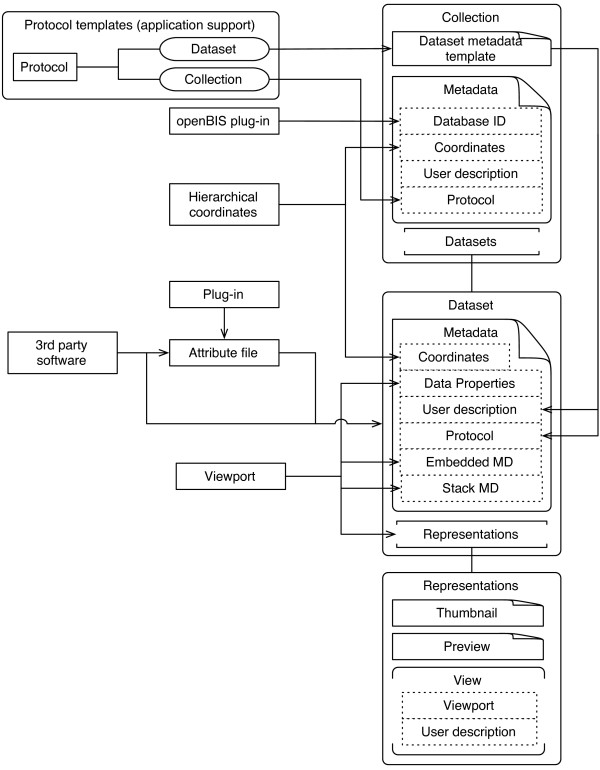
**Metadata system of openBEB.** All metadata for the collection and the associated raw-data are stored in the annotation folder of a collection. See text for details.

The metadata handling system is an important aspect of openBEB. Every collection and dataset contains separate metadata. The XML-based metadata files are organized in several sections (Figure 4): (i) Protocols; provide domain and technique specific information. Protocol templates based on a controlled vocabulary are organized centrally in the application support folder and are automatically updated during program start-up. (ii) User descriptions; contain additional user information, such as free text annotations and a rating system. (iii) Database ID (collection only) and coordinates; indicate the relationship between collections and the data sets they contain. The hierarchical coordinate system (see below for details) defines the physical relationship between different experiments and datasets in space and time (if needed). (iv) Dataset properties (see below).

**Figure 5:**
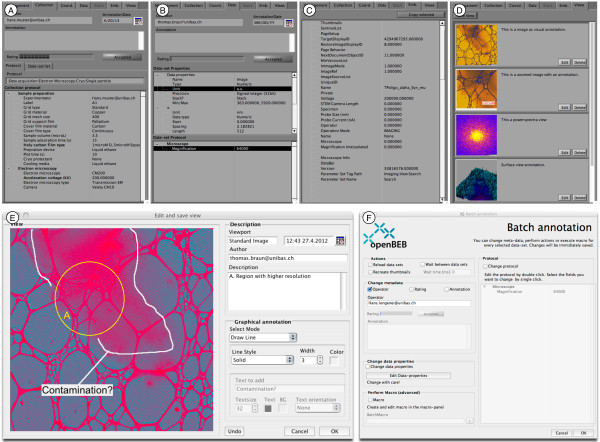
**Metadata subsystem GUI of openBEB.** Data can be annotated by pre-defined “protocols” **(A and B)** and graphically **(D and E)**. **A)** Collection metadata. **B)** Dataset metadata. **C)** Embedded metadata of the raw-data file, here the embedded information of a Gatan dm3 file. **D)** Views list: “Views” of the graphical representations of each file in a dataset. **E)** User interface to create and annotate views; graphical annotation is possible. **F)** User interface to perform batch annotations.

In addition, openBEB supports graphical annotations by creating portable network graphics files (png). This metadata includes representations required for each dataset; both a thumbnail and a preview file are created. Further, graphically annotated views can be stored (see Results and discussion, Figure 5).

Metadata entries can be added in various ways, e.g., by using the GUI (see Results and discussion). Automatic annotation is of importance for high throughput measurements and can be performed either by directly accessing the XML metadata-files through openBEB, or by writing an attribute file to accompany the raw data. The latter allows the transfer of embedded metadata information from the raw-data file into the explicit metadata of a dataset.

The metadata file also contains a description of the *dataset properties*. These data properties are very flexible and support an arbitrary number of dimensions. They are designed to describe equally spaced data, but are not limited to this. A *dimension* contains a name, a type (e.g., numeric or index), a unit, a start and spacing (only for equally spaced data) and a length (number of data points in the direction of the axes). The data elements themselves are described by the data-type; consisting of a name, unit and type (e.g., numeric or category) and a precision that indicates the memory structure of the data, e.g., 64 bit floating point.

**Figure 6:**
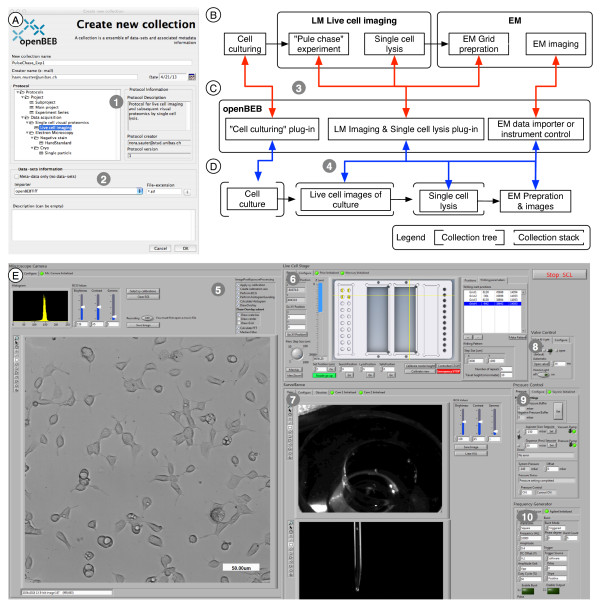
**Creation of a new collection, instrument control and data-acquisition. A)** Dialog to create a new collection. Importantly, a collection protocol (1) must be chosen, and an importer can be selected if needed (2). **B)** Example workflow for biological experiments and single cell “lyse and spread” visual proteomics. **C)** openBEB plug-ins employed to control the instrument, perform time-lapse imaging of the cells, prepare samples from individual cells for EM (i.e., dialysis/staining and deposition on EM grid) and subsequently import EM images for analysis by visual proteomics. **D)** Tree-like structure of collections containing datasets and metadata created by openBEB. The relationship between the workflow and openBEB components is indicated by red arrows (3). The blue arrows (4) show the relationship between the openBEB components and the collection-tree. Note that the cell culture LM images define a coordinate system, and the coordinates of subsequent EM images define a sub-coordinate system (HC system, see text). Instrument control and data acquisition plug-in combining LM and single cell lysis. This plug-in contains different modules communicating via the openBEB macro system. (5) Live cell imaging. (6) Stage control. (7) Control optics. (8 to 10) Pump-system and electroporation control.

The coordinate system is hierarchical (see Table 1) and allows the correlation of different experiments, each measuring a specific property of the same subject. Collections or datasets can define the root of a coordinate hierarchy, or can be at a specific point in the coordinate system relative to the parent collection, e.g., using a different scale (see description in Table 1). Note: the coordinate systems are linear and the coordinate vectors are additive (illustrated in the Results and discussion, Figure 6). An internal timer that uses an NTP (network time protocol) client for calibration provides a clock for absolute time stamps. As long as the same NTP server is used, this allows the synchronization of measurements made using different instruments.

**Table 1:** Hierarchical Coordinate (HC) fields

**Parameter**	**Description**
Rootflag	Boolean. True if this entity is the coordinate system root.
x, y, z	Cartesian. Internally the coordinates are stored as picometer (pm) values in a signed 64-bit integer. These coordinates are relative to the parent coordinates (i.e., for collections, the parent collection; for datasets, the containing collection).
Scale	Multiplication factor for Cartesian coordinates; for better readability, e.g., set the scale to 1000 if nanometer values should be displayed.
dx, dy, dz	Estimated standard error of Cartesian coordinates (in pm).
t	Time stamp in seconds since 1.1.1904 00:00:00 Universal Time ignoring leap seconds. The time stamp is represented in a 128-bit fixed-point number with a 64-bit radix. The first 64 bits are a signed integer for the seconds, the second 64 bits are unsigned fractions of a second.
dt	Estimated standard error of the time stamp (in fractional seconds).
Description	Description of the coordinate system (string); field to provide additional, ‘human readable’ information.

Fields that are not applicable must be described as NaN (not a number). Error estimates can be provided for every parameter.

#### Error handling

**Figure 7:**
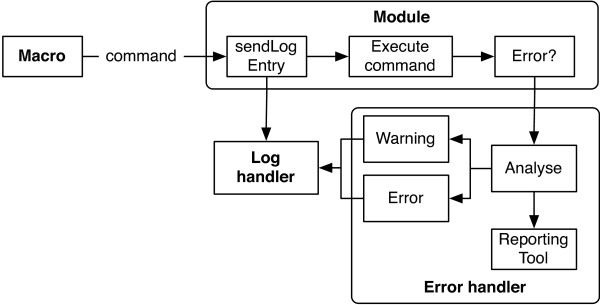
**Error and logging subsystem of the openBEB core program.** The individual modules receive commands from the macro-parser and execute them in a ‘first in first out’ order (queued state machine). Immediately before execution, the command is sent to the log handler, which writes it directly to the log file. After execution, if a warning or error occurred the module sends an error report to the error handler. Optionally, a reporting tool is triggered for error reporting. A log file viewer is provided in the form of a plug-in.

OpenBEB implements centralized, system-wide error handling (Figure 7). A log server maintains a log of all events taking place in all running modules, i.e., in both the core program and the plug-ins. A central error handler reacts to errors. Optionally, errors can be reported to an issue tracker and viewed via a GUI. Different levels of error tolerance can be set to allow automation.

### Plug-in manager

The experiment-specific functionality of openBEB is provided by plug-ins. A plug-in can provide functionality to the core program in two ways: (i) By using functions to override standard core routines, e.g., importing a dataset by an importer. (ii) By using so called “modules”, i.e., a queued state machine that can be addressed by the macro interpreter as described above. Plug-ins are instantiated during program start-up and destructed when openBEB quits. Before loading, a plugin.ini file is read to confirm compatibility and provide plug-in descriptions for the user. The plug-in is then initialized, e.g., standard settings are read from the file-system or plug-in-specific modules are started. Note that either plug-ins that function as stand-alone programs or plug-ins that call other 3^rd^ party software can be written for instrument control. The homepage of openBEB provides specific information and tutorials on how to write plug-ins. The available plug-ins are distributed together with their source code and can thus easily be studied or changed locally.

**Figure 8:**
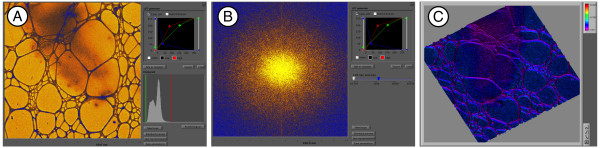
**Different viewports visualizing the same image. A)** Standard view of the image in real-space. A look-up color table has been added and can be adjusted (top right), as can the greyscale histogram (bottom right). **B)** Power spectrum of the image. **C)** Surface view of the image. The different views can be saved as new files at will and are thus called “views” (see Figure 5D). Note that you can easily switch between the different views (Figure 13, B5).

The openBEB plug-ins are organized hierarchically (Table 2): (i) ViewportType plug-ins are libraries providing data-type definitions (e.g., for images, or time resolved “wave” data). (ii) Library plug-ins do not provide data-type definitions but basic functionalities, such as data-manipulation routines or basic instrument control functionalities. Other plug-ins can be dependent on viewportTypes or libraries. (iii) Viewports are plug-ins for data visualization. They are always linked to a data-definition library and provide a module called “viewport” which must understand specific commands such as ‘display data’ or ‘unload data’. Note that every data-definition library can have several linked viewports allowing the visual representation of different aspects of a data type, e.g., the display of an image in real or Fourier space (demonstrated in Results and discussion, Figure 8).

**Table 2:** List of plug-in types

**Type**	**Dependency**	**Description**
ViewportType		Provides a data-type definition and also a library containing routines allowing specific manipulations to be carried out for the defined data-type. Are loaded first.
Library	(ViewportType)	Libraries; can be dependent on ViewportTypes. Are loaded immediately after ViewportType definitions.
Viewport	ViewportType	Data visualization plug-ins. A specific data-type (defined in the viewportType) can have multiple viewports. Viewports are presented in the main window (see Figure 13A).
Collection	(ViewportType, Library)	Plug-in for data import or instrument control.
Importer	ViewportType, (Library)	Imports data.
Tool	(ViewportType, Library)	Small subprogram. Can be invoked with the tool menu in the main window (Figure 13B).
Database	(ViewportType, Library)	Plug-in for database connection. The standard plug-in synchronized with openBIS [6].
Module	(ViewportType, Library)	Plug-in providing a module (commands) for the macro-interpreter.

The plug-ins are loaded during program start-up, the position in the table indicates the load order. (): optional.

**Figure 9:**
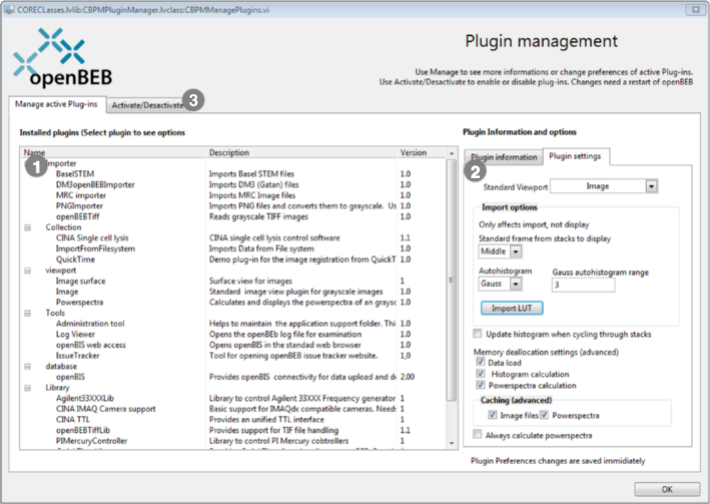
**Plug-in manager of the core program.** When the ‘Manage active Plug-ins’ subpanel is selected, a list of all active plug-ins is shown on the left (1). The plug-in information panel and a GUI for the plug-in specific settings are displayed when a plug-in is selected (2). Installed plug-ins can be activated or deactivated using the second subpanel (3).

The plug-in manager (Figure 9) displays information about all installed plug-ins and allows them to be activated/deactivated at will (needs an openBEB restart). Every plug-in also owns a preferences GUI that can be accessed by the plug-in manager.

## Results and discussion

The use of openBEB is first demonstrated for administrators and plug-in developers. A typical scenario describing how openBEB is used to combine live cell microscopy, microfluidic control and a new approach for visual proteomics called “spread and lyse” [8,9], is then presented.

### Installation, administration and plug-in development

#### Installation and update

**Figure 10:**
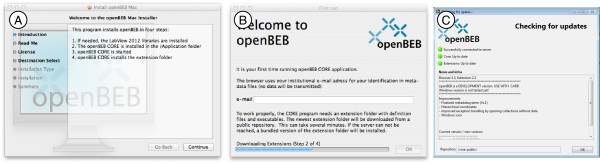
**Installation, first run and update. A)** Standard OS X installer to install the core program and required runtime libraries. A similar installer is provided for Windows. **B)** First run; user interface installing the application support folder. **C)** User interface of the update procedure during start-up. The application support folder is automatically updated without user interaction. A control mechanism checks whether the current openBEB application is compatible with the updated application support folder, if not, the newest openBEB core program is downloaded and installed (needs administration rights).

**Figure 11:**
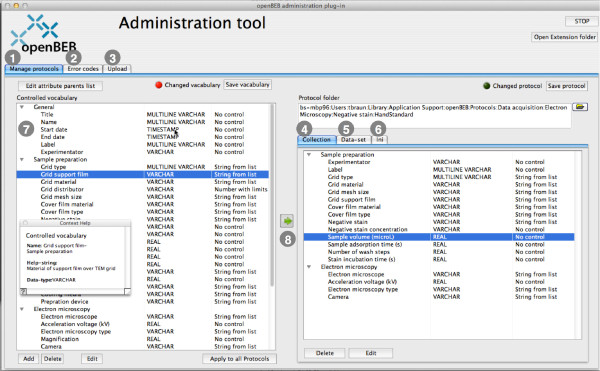
**Administration tool and protocol management.** The administration plug-in manages the application support folder. The tool allows the maintenance and creation of protocols (1), the definition of openBEB errors (2) and the semiautomatic upload of the application support folder to the server (3). Protocols are comprised of three files: a collection protocol (4), a dataset protocol (5), and an initialization file (6). The first two are assembled from a library of controlled vocabulary items (7) that is shared between all protocols. An entry in the controlled-vocabulary library can be changed, and these modifications can be automatically transferred to existing protocols (8).

OpenBEB is installed in two steps: First, a standard installer (Mac or Windows) installs the runtime library and the core program (Figure 10). Second, the core program downloads and installs the application *support folder* containing the plug-ins, metadata template definitions, macros and libraries. This folder is different for every user and is located in a directory with full user access. During openBEB start-up, the application support folder is updated to the newest version from a server-side repository (Figure 10). This allows the metadata templates and plug-ins to be updated in a centralized way for a workgroup. Different repositories can be specified for different work environments. An administration plug-in facilitates the management of the application support folder (see below and Figure 11).

#### Protocol management

Protocols are managed by a GUI provided by the administration plug-in (Figure 11). Protocols are created in two steps: First, a controlled vocabulary library is defined; the same vocabulary is used for all protocols. Second, the protocols are assembled from the controlled vocabulary. An entry in a protocol can be modified; the parent title can be changed as well the standard value of the entry. Note, that every vocabulary entry is associated with a unique identifier, which allows protocols to be automatically updated if a controlled vocabulary entry is changed.

#### Plug-in development

**Figure 12:**
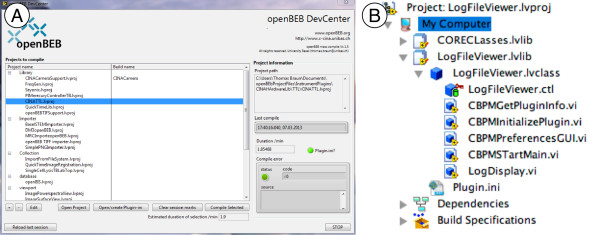
**Development of openBEB plug-ins. A)** openBEB DevCenter facilitating management of the LabView project file created for each plug-in and maintenance of the plugin.ini file that provides essential information about the plug-in. The tool also allows the batch-compilation of selected plug-ins. **B)** Typical layout of a plug-in in the LabView project manager. Every plug-in provides a minimal set of override functions: “BPMGetPlugin” info provides information about the plug-in, “CBPMInitialize plug-in” initializes the plug-in during start-up, “preferences GUI” provides a GUI to manage plug-in settings, “CBPMStartMain” starts the main plug-in program.

Templates and tutorials are provided on the software homepage http://www.openBEB.org. A helper program called “openBEB DevCenter” (openBEB development center) that facilitates the batch compilation of plug-ins and editing of the plug-in initialization file, is also downloadable (Figure 12).

To create a plug-in, a LabView project is created including the openBEB core and any other openBEB library required. A plug-in must provide a minimal set of override functions, which are called during execution. The specific functions that must be present depends on the type of plug-in (see the development section of the openBEB software home-page).

### Example: Live cell imagining and “Lyse and spread” visual proteomics

The use of openBEB together with our recent hardware developments that connect micro-fluidics to EM for visual proteomics [8,10] is reported. In the demonstration experiment adherent eukaryotic cells are cultured, and monitored by time-lapse LM throughout. At specific time points, pulse chase investigations are performed and, after a chase time, individual cells are lysed by electroporation [10]. Subsequently, the cell lysate is transferred into a microcapillary, prepared for EM and imaged for analysis by “lyse and spread visual proteomics” [8,9]. The presentation focuses on the application of openBEB using the GUI, and demonstrates some typical aspects of the program.

#### Main window of the openBEB GUI

**Figure 13:**
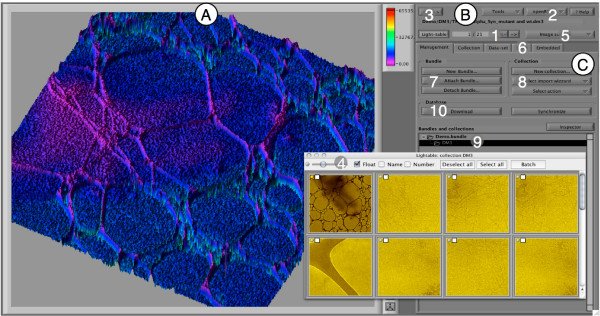
**Main window of the openBEB browser.** The main window can be used as a single window or in full-screen mode. The window has three parts: **A)** Viewport for data visualization. These viewports are dynamically opened when browsing datasets. **B)** Tools and navigation panel. **C)** Management and metadata panel. (1) Navigation for dataset browsing. (2) Tools and openBEB menu pop-up’s. (3) Full-screen toggle. (4) Light-table window, showing small thumbnails of all datasets in the open collection (can be opened and closed). (5) Viewport selection; a specific data-type can be visualized by viewports. (6) Metadata-panels for collections and datasets. (7) Bundle management. (8) Collection management. (9) Tree representation of attached bundles (“root collection”) and collections. (10) User interface triggering database synchronization or download of data.

OpenBEB can be run headless and controlled via the *application serve*r, or by a GUI (Figure 13). The main window of the GUI has three panels namely the *viewport* (Figure 13A), the *tools and navigation* panel (Figure 13B) and the *management and metadata panel* for core program control (Figure 13C).

#### Creating and managing collections, instrument control and data acquisition

Bundles and collections are managed using the management panel of the metadata tab control (Figure 13, C7 and 8). Figure 6 depicts the user interface that allows a new collection to be initiated (A). A typical workflow combining cell culturing, time-lapse LM imaging and visual proteomics by EM is shown (B). The responsible plug-ins are indicated (C), and the resulting collection hierarchy is depicted (D). The plug-in controlling live cell imaging and sample preparation for EM, is shown as an example (E).

Note, coordination between the different measurement domains is crucial, and is achieved by the HC system: As an example, the cell culture defines a (root) coordinate system in x and y and the LM images can be calibrated accordingly. Subsequently, an individual cell imaged by LM is selected for further experiments, e.g., lysis. The new data is stored in a sub-collection (e.g., Single cell lysis, Figure 6D). The data from this analysis define a sub-coordinate system; this sub-coordinate system depends on the root coordinate data of the mother collection (here the cell culture). Together, this defines a HC system as described above. In other words: To find the soccer stadium, latitude and longitude “GPS” coordinates are reasonable, to find a player on the soccer field, a rectangular coordinate system with the origin at a corner is better suited. The two systems define a HC system.

#### Data browsing and visualization

Various viewport plug-ins are available for data visualization. The type required depends on the data-type. Data types are defined in so-called viewportType plug-ins (Table 2).

Three viewport plug-ins are currently provided for the visualization of scientific greyscale images (Figure 8A-C). The first visualizes images in real space and allows the addition and adjustment of a look-up table as well as the visualization of the greyscale histogram (A). The second calculates and displays the power-spectrum of the image data. This is useful to evaluate the contrast-transfer function of the imaging device (B). The third uses the data values as height information and draws an interactive surface plot representation (C). Note, that viewport data visualization profits from the rich libraries provided by the LabView environment, facilitating the development of such plug-ins.

#### Data annotation and metadata management

OpenBEB includes a comprehensive metadata management tool. Metadata can be assigned to collections, datasets and, depending on the data-type, to frames of a dataset stack, e.g., time series. Annotations can be created or modified using the GUI (Figure 5A-F). Individual annotations can be written to collections (A) and dataset files (B). If supported by the importer, the metadata embedded in the raw-data file is displayed (C). Furthermore, the data visualization provided by the different viewports can be stored as “views” (D) that can also be graphically annotated if required (E). A batch-annotation tool facilitates changes to the metadata of selected data series (F).

#### Database synchronization with openBIS

OpenBEB supports database synchronization plug-ins; a standard plug-in to synchronize with an openBIS database is presented here. This plug-in lets openBEB transparently store a user’s local work on a server, which has several benefits: (i) It makes the user’s work accessible to other team members or collaborators. (ii) It protects against data loss. Having a second copy of the data on a server enables data recovery in the event of hardware failure or loss. The server, which is managed by a system administrator, is typically configured to make a nightly back up. (iii) Synchronization with a server ensures that work remains accessible beyond the duration of a PhD (unfortunately still the typical lifetime of scientific data). Storing the data on a server, along with any required contextual metadata, makes it possible for future team members to understand and build upon the results of present or previous team members. (iv) It further increases the scalability of the overall system as only recent data has to be kept locally in openBEB, while older data can be offloaded to openBIS.

**Figure 14:**
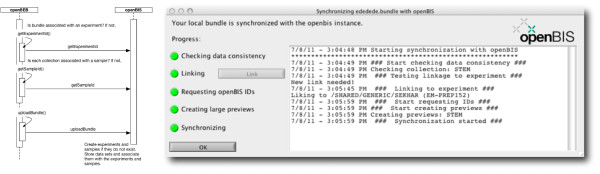
**Communication diagram explaining and illustrating the uploading of an openBEB bundle to openBIS [**[6]**].**

The data synchronization plug-in for openBIS is a standard plug-in supplied with openBEB (Figure 14). The openBIS data synchronization plug-in stores the raw-data and the accompanying metadata in openBIS. Just pushing the data to the server is not sufficient; information about the experiment that produced the data and the biological and technical samples that were measured is required as well. To reinforce this connection, the openBEB synchronization process creates an experiment and sample in openBIS. Information about the experiment and sample are important for data-provenance tracking.

### Limitations and comparison to other software

The functionality of openBEB depends on the available plug-ins. Currently, two viewportType families are provided, each defining a data-type; one is for greyscale images (as presented above), the other is for “wave” data providing support for equally spaced measurements, e.g., a time resolved signal. Further, to a certain degree available plug-ins for hardware control are inherently tied to the hardware and communication protocols of a local set-up. Accordingly, the instrument and data-acquisition control system presented in Figure 6 is directly linked to specific hardware developed in-house, and limited to this specific setup. However, the extensive libraries for data acquisition, processing and visualization of LabView make it easy to develop new plug-ins, and openBEB hardware integration via library plug-ins allows a high degree of code re-usage. Furthermore, the core system of openBEB facilitates the integration of independently developed modules.

To our knowledge, openBEB is the only data-agnostic browser that has both developers and the end-user in mind. It complements “single domain” software solutions [4,5]. Use of openBEB can provide the unified control and tight integration essential to maintain the temporospatial relationship of correlated experiments. Combining the data-type independent housekeeping tools offered by the openBEB core program with the flexibility of the plug-in system makes this possible.

## Conclusions

OpenBEB is a tool for correlative experiments in systems biology. It allows instrument control and provides a bridge between biological experiments, annotation and synchronization with databases. The software is (i) for end-users performing automated (biological) experiments and (ii) for developers requiring a framework to write instrument control and data-registration plug-ins. The latter inherently benefit from the “house-keeping” data-management and annotation tools of the openBEB core program. The plug-in based, loose coupling and the open modular architecture of the macro subsystem make openBEB highly flexible and scalable. Further data-type plug-ins will be implemented in the future, e.g., to directly access mass-spectrometry data.

## Availability and requirements

The openBEB core program has the following requirements and is available as indicated:

**Project name:** openBEB

**Project home page:**http://www.openBEB.org

**Operating system(s):** Mac OS X, Windows XP, 7; will be compiled on Linux

**Programming language:** G (LabView), JAVA

**Other requirements:** LabView runtime 2012, JAVA. Minimal screen-size of 1024 × 720 pixel

**License:** Apache license (http://www.apache.org/licenses/LICENSE-2.0)

**Any restrictions to use by non-academics:** no restriction, however restrictions may apply for specific plug-ins.

## Abbreviations

openBIS: open Biological Information System; openBEB: open Biological Experiment Browser; GUI: Graphical user interface; HC: Hierarchical coordinate system; EM: Electron microscopy; LM: Light microscopy; NTP: Network time protocol.

## Competing interests

The authors declare that they have no competing interests.

## Authors’ contributions

TB developed the software concept and wrote most of the core software and plug-ins; NS developed the instrument plug-in connecting live cell imaging, microfluidic based cell lysis and sample preparation for visual proteomics (shown as an example); CR, KE and BR critically reviewed openBEB progress, developed/maintained openBIS and created the openBEB-openBIS bridge; AB, SR, PR, KNG, HS, SAM tested the software and/or provided significant feedback during the software development. All authors were involved in the manuscript preparation. All authors read and approved the final manuscript.
